# Glucose-Dependent Insulinotropic Polypeptide Plasma Level Influences the Effect of n-3 PUFA Supplementation

**DOI:** 10.3390/diagnostics12081984

**Published:** 2022-08-16

**Authors:** Joanna Goralska, Urszula Razny, Philip C. Calder, Anna Gruca, Caroline E. Childs, Piotr Zabielski, Aldona Dembinska-Kiec, Maciej Banach, Bogdan Solnica, Malgorzata Malczewska-Malec

**Affiliations:** 1 Department of Clinical Biochemistry, Jagiellonian University Medical College, Skawinska 8, 31-066 Krakow, Poland; 2 School of Human Development and Health, Faculty of Medicine, University of Southampton, Tremona Road, Southampton SO16 6YD, UK; 3 Department of Medical Biology, Medical University of Bialystok, 2C Mickiewicza Street, 15-222 Bialystok, Poland; 4 Department of Preventive Cardiology and Lipidology, Medical University of Lodz (MUL), Rzgowska 281/289, 93-338 Lodz, Poland

**Keywords:** glucose-dependent insulinotropic polypeptide, GIP, incretin, gut hormone, n-3 PUFA, DHA, EPA, supplementation, fatty acids

## Abstract

Elevated glucose-dependent insulinotropic peptide (GIP) levels in obesity may predict the metabolic benefits of n-3 PUFA supplementation. This placebo-controlled trial aimed to analyze fasting and postprandial GIP response to 3-month n-3 PUFA supplementation (1.8 g/d; DHA:EPA, 5:1) along with caloric restriction (1200–1500 kcal/d) in obese subjects. Compliance was confirmed by the incorporation of DHA and EPA into red blood cells (RBCs). Blood analyses of glucose, insulin, non-esterified fatty acids (NEFAs), GIP and triglycerides were performed at fasting, and during an oral glucose tolerance test and a high fat mixed-meal tolerance test. Fatty acid composition of RBC was assessed by gas chromatography and total plasma fatty acid content and composition was measured by gas–liquid chromatography. The DHA and EPA content in RBCs significantly increased due to n-3 PUFA supplementation vs. placebo (77% vs. −3%, respectively). N-3 PUFA supplementation improved glucose tolerance and decreased circulating NEFA levels (0.750 vs. 0.615 mmol/L), as well as decreasing plasma saturated (1390 vs. 1001 µg/mL) and monounsaturated (1135 vs. 790 µg/mL) fatty acids in patients with relatively high GIP levels. The effects of n-3 PUFAs were associated with the normalization of fasting (47 vs. 36 pg/mL) and postprandial GIP levels. Obese patients with elevated endogenous GIP could be a target group for n-3 PUFA supplementation in order to achieve effects that obese patients without GIP disturbances can achieve with only caloric restriction.

## 1. Introduction

Obesity is closely associated with metabolic complications such as insulin resistance, dyslipidemia, hepatosteatosis and chronic inflammation. Glucose-dependent insulinotropic polypeptide (GIP) seems to play an important role in all these areas of dysmetabolism. Produced by the enteroendocrine K-cells in the duodenum and secreted into the circulation in response to the ingestion of meal, GIP stimulates glucose-dependent insulin secretion. Recently, other pleiotropic effects of GIP have been reported, such as roles in postprandial lipid homeostasis, bone turnover, energy balance and adiposity, which results from discovering the presence of functional GIP receptor (GIPR) in various tissues, including adipose tissue and the brain [1,2,3]. Some obese patients have relatively high plasma GIP levels, which may reflect a compensatory mechanism in order to overcome the diminished islet response. Hypersecretion of GIP along with its impaired insulinotropic action is common in people with obesity and diabetes mellitus [4,5].

Three months of supplementation with the combination of the n-3 polyunsaturated fatty acids (n-3 PUFAs) eicosapentaenoic acid (EPA) and docosahexaenoic acid (DHA) at a combined dose of 1.8 g/day was reported to modulate obesity-related disorders, such as inflammation and dyslipidemia and to prevent loss of bone health [6,7]. Many studies and clinical investigations have addressed the beneficial effects of n-3 PUFAs. Pooling of several global studies using biomarkers of n-3 PUFAs in participants without prevalent coronary heart disease (CHD) revealed that high status of n-3 PUFAs is associated to reduced risk of fatal CHD [8]. The beneficial effects of the main bioactive n-3 PUFAs, EPA, docosapentaenoic acid (DPA) and DHA, on lowering cardiovascular disease (CVD) risk are related to antiatherogenic effects and/or to the modification of risk factors through mechanisms related to lipid metabolism. Several trials demonstrate that supplementation with EPA and DHA can reduce circulating triglyceride levels as well as liver fat [9,10,11,12,13]. The mechanisms involve the decrease in hepatic de novo lipogenesis and the partitioning of fatty acids away from triglyceride synthesis and toward β-oxidation [14].

Our previous data indicate the association of plasma GIP levels not only with glucose metabolism but also fasting lipids, chronic inflammation, and liver function [15,16]. The mechanism underlying associations of GIP with lipid metabolism warrants further study, especially in the light of the development of new incretin-based pharmacotherapies for the treatment of obesity, dual GIP and GLP-1 receptor agonists.

The study was aimed at identifying the fasting and postprandial GIP responses to 3-months intervention with n-3 PUFAs along with caloric restriction in obese subjects. In the second step, effects of the intervention on the plasma fatty acid profile were assessed in two groups according to plasma GIP level. This approach was designed to answer the question of whether circulating GIP status could modulate the response to n-3 PUFA supplementation. An analysis of the lipidomic data from our trial indicates that n-3 PUFA supplementation significantly improves the plasma fatty acid profile in obese patients with high GIP plasma levels compared to those with lower GIP levels. This led us to raise a new hypothesis that obese patients with impaired endogenous GIP secretion / signaling could be a target group for n-3 PUFA supplementation, to achieve effects that obese patients without GIP disturbances can achieve with only caloric restriction.

## 2. Materials and Methods

### 2.1. Study Design and Subjects

Subjects aged 25–65 years, with abdominal obesity (waist circumference > 102 cm in men and >88 cm in women) and BMI in the range 25–40 kg/m^2^ were eligible to participate in the trial. Exclusion criteria were diabetes, dyslipidemias, endocrine, metabolic, or chronic inflammatory diseases, kidney or liver dysfunction. Participants on lipid-lowering or anti-inflammatory drugs or supplements containing vitamins or PUFAs were excluded. Fish consumption was not allowed during the study period.

This 3-month clinical trial was randomized, double-blind, placebo-controlled parallel and single center. The trial was approved by the Bioethics Committee of the Jagiellonian University in Krakow (written consent, opinion No. KBET/82/B/2009) and all participants provided written informed consent. The trial was carried out at the Department of Clinical Biochemistry, Jagiellonian University Medical College, Krakow, Poland, in accordance with Declaration of Helsinki and with the Good Clinical Practice guidelines. The clinical study was registered at isrctn.com as ISRCTN11445521.

**Figure 1:**
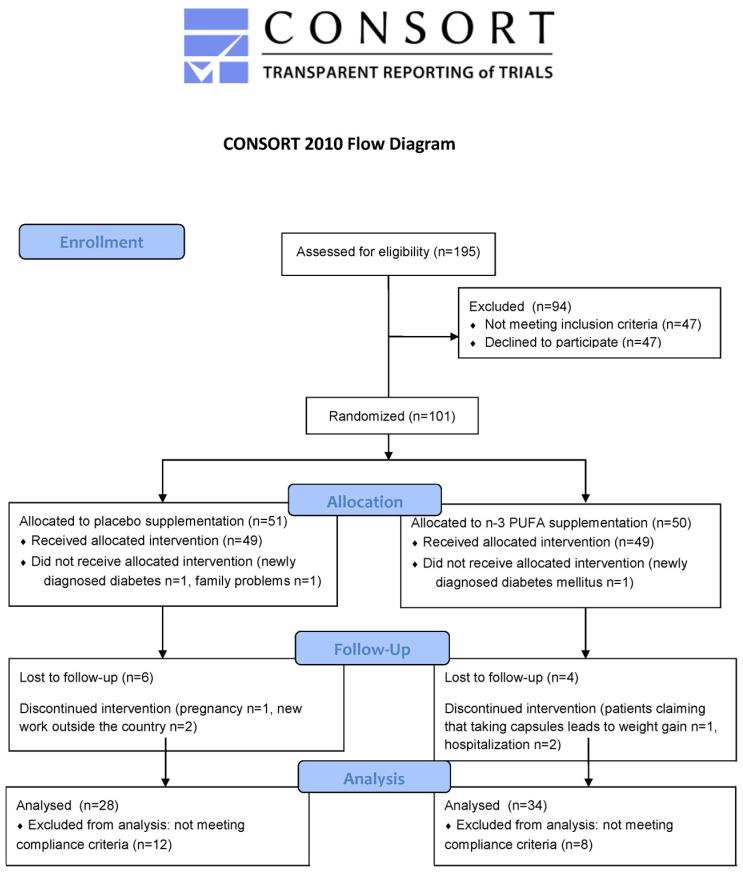
CONSORT flow diagram.

All recruited participants had a detailed medical examination, and BMI and waist circumference were measured to screen for eligibility; standard laboratory tests were performed to evaluate health status and to exclude volunteers who met any exclusion criterion. All of the participants that enrolled into the study underwent a 2-week adaptation period to a diet containing the amount of calories according to individual caloric requirement, which was calculated by a nutritionist considering the basal metabolic rate and the level of physical activity of the participant. This isocaloric diet (2300–2400 kcal/day) contained 57% of energy from carbohydrates, 30% from fat and 13% from protein. Participants were advised on how to apply this diet and were given written instructions as support. After the adaptation period, participants were randomly assigned to the n-3 PUFA or placebo groups using an independent online computerized randomization system by a member of administrative staff who was not involved in recruitment, clinical care, or dietary advice. Randomization was conducted at the level of the individual and was stratified by sex and age (Figure 1).

Participants were advised to adopt a low-calorie diet (1200 kcal/day for women and 1500 kcal/day for men) in which 60% of energy was provided from carbohydrates with low glycemic index, 15% from protein and 25% from polyunsaturated fat. Participants assigned to the calorie restricted diet supplemented with n-3 PUFAs (CR + n-3 PUFAs group) were additionally given capsules with n-3 PUFAs (EPAX 1050 TG; EPAX, Alesund, Norway) to consume 3 times per day immediately after a meal. Participants assigned to the calorie restricted diet supplemented with placebo (CR + placebo) were additionally given capsules with corn oil to consume 3 times per day immediately after a meal. Both placebo and n-3 PUFA capsules contained 4 mg of vitamin E per capsule and were identical in size, shape, and appearance to ensure the blinding of participants and nutritionists. One n-3 PUFA capsule contained 0.6 g docosahexaenoic acid (DHA) + eicosapentaenoic acid (EPA) in a ratio of 5:1. The amount of 1.8 g DHA + EPA daily is considered safe and is equivalent to 4 servings of oily fish per week. During the implementation of the 3-month intervention, all participants were under the supervision of a dietitian with visits every two weeks.

To assess the compliance to n-3 PUFA supplementation, besides counting returned capsules, the fatty acid composition of plasma as well as DHA and EPA content in red blood cell (RBC) membranes were measured before and after 3-month intervention. An increase by minimum 1 in omega-3 index, which is the sum of EPA and DHA in RBC membranes, was used to identify compliers in the n-3 PUFA group and noncompliers in the placebo group. Finally, the analyses were conducted for 28 participants in the placebo group and 34 in n-3 PUFA group, all of which met the compliance criteria (Figure 1).

### 2.2. Anthropometric Measures and Blood Collection

At baseline (after 2 weeks of adaptation period) and at the end of 3 months intervention, anthropometric measurements were made, blood was collected at fasting as well as at 5 time points of an oral glucose tolerance test (OGTT) and at 5 time points of a high fat mixed meal tolerance test (HFMTT).

Anthropometric measurements included body weight, height, BMI, waist and hip circumference and adipose tissue content (Tanita Body Composition Analyser BC-418, Tanita, Tokyo, Japan).

Participants were instructed to avoid strenuous exercise and alcohol consumption the day before blood collection. After a 12 h overnight fast, venous blood was collected, centrifuged (1000× *g* for 10 min at 4 °C within 30 min from collection) and serum, plasma and RBC samples were immediately frozen and stored at −80 °C for further analyses of glucose, insulin, GIP, total cholesterol, high density lipoprotein-cholesterol (HDL-cholesterol), low density lipoprotein-cholesterol (LDL-cholesterol), triglycerides (TGs), and total plasma fatty acid content and composition.

Oral glucose tolerance test (OGTT) and high fat mixed meal tolerance test (HFMTT) were performed on separate days. Venous blood samples: fasting, 30, 60, 90 and 120 min of OGTT as well as fasting, 2, 4, 6, and 8 h of HFMTT were collected in order to measure postprandial glucose, insulin, GIP, non-esterified fatty acid (NEFA) and TG concentrations. An 8-h HFMTT that contained 73% fat, 16% protein, and 11% carbohydrates, with a caloric value of 1018 kcal, was performed. The detailed composition of the meal has been described previously [17].

### 2.3. Blood Analyses

Plasma GIP level was measured using ELISA (EMD Millipore, St. Charles, MO, USA). Within-run CV was 6.1%, between-run CV 8.8% and the limit of detection was 8.2 pg/mL. Plasma glucose, total cholesterol, HDL-cholesterol, and TGs were assayed by automated, enzymatic colorimetric methods (ELITech Clinical Systems, Sées, France). The intra- and inter-assay variability coefficients were as follows: 2.3% and 3.5% (glucose), 1.4% and 3.4% (TGs), 1.4% and 3.8% (total cholesterol), 2.1% and 2.8% (HDL-cholesterol), respectively. LDL-cholesterol was calculated according to the Friedewald formula. Insulin was determined by an immunoradiometric method (DIAsource ImmunoAssays, Ottignies-Louvain-la-Neuve, Belgium) and read using a gamma counter (LKB Instruments, Mount Waverley, Victoria, Australia); within and between-run imprecision CVs were 2.1% and 6.5%, respectively. NEFA concentration was measured in fresh serum by an enzymatic quantitative colorimetric method (Roche Diagnostics GmbH, Mannheim, Germany).

The fatty acid composition of RBCs was assessed by gas chromatography using methods described previously [18]. Chloroform/methanol (2:1 *v/v*) was used to extract total lipid. RBC lipids were saponified: in the presence of sulfuric acid containing 2% methanol, fatty acid methyl esters were formed by heating at 50 °C for 2 h, extracted into hexane and then concentrated by evaporation under nitrogen. Separation of fatty acid methyl esters was performed by gas chromatography on a Hewlett Packard 6890 gas chromatograph (Hewlett Packard, Palo Alto, CA, USA) fitted with a BPX70 column (SGE Europe, Milton Keynes, Bucks, UK). Run conditions are described elsewhere [18]. Identification of fatty acids was by comparison of their retention times with those of authentic standards. Data are presented as percentage contribution to the total fatty acid pool. Omega-3 index was calculated as EPA + DHA.

Total plasma fatty acid content and composition was measured by gas–liquid chromatography and flame-ionization detector on Hewlett Packard 5890 Series II gas chromatograph, fitted with CP-Sil 88 FAME column, after direct in situ transesterification, according to Glaser et al. [19]. The plasma fatty acid profile included quantitative determination of saturated (myristic, palmitic, stearic, behenic, lignoceric, and arachidic), monounsaturated (palmitoleic, oleic and nervonic) and polyunsaturated (arachidonic, linoleic, α-linolenic, eicosapentaenoic, and docosahexaenoic) fatty acids.

### 2.4. Data Analysis

Nominal data were analyzed by chi-square (χ^2^) test and presented as number and percentage. Continuous variables are presented as mean ± SD. Levene’s test was used to check for the homogeneity of variance. The Shapiro-Wilk test was used to check for normal distribution and data were transformed if required to obtain normal distribution for further analyses. The differences between the two study groups were analyzed by Student’s *t*-test. Differences between two time points within a group were analyzed by paired *t*-tests. In the secondary analysis, subjects were divided into two subgroups according to fasting GIP plasma level, using the median (29 pg/mL) as cut-off point. ANOVA with repeated measures and post-hoc tests (Tukey and Bonferroni) was used to evaluate the time effect, type of supplementation effect, GIP group effect, as well as interactions between these factors. *p* values < 0.05 were considered significant. Statistical analyses were performed with Statistica software v.13.3 (StatSoft).

## 3. Results

### 3.1. Participant Characteristics

Participants assigned to placebo or n-3 PUFA supplementation groups did not differ at baseline in terms of anthropometrics, age, sex, and basic biochemical variables (Table 1). Carbohydrate metabolism markers, such as fasting glucose and insulin levels were similar in both groups as well as lipid profile, fasting NEFAs and the plasma concentration of GIP (Table 1).

### 3.2. Effect of n-3 PUFA Supplementation on Markers of Carbohydrate and Lipid Metabolism

After a 3-month moderate caloric restriction, the loss of weight, BMI and body fat was independent of the supplementation group (Table 2). Nevertheless, only n-3 PUFA supplementation resulted in significant increase in EPA and DHA content in plasma as well as in RBC membranes. Omega-3 index, which is the best indicator of sustainable n-3 PUFA status, increased by 77% exclusively in the n-3 PUFA supplemented group. Plasma levels of DHA (increase by 78%) and EPA (increase by 62%) reflected very closely the effect of supplementation on RBC membranes. Among participants who had a baseline omega-3 index below 4%, after n-3 PUFA supplementation none stayed in this omega-3 index zone (4 pre vs. 0 post), whereas after placebo supplementation most participants remained in this zone (5 pre vs. 4 post). Moreover, the number of participants who presented relatively high (>8%) omega-3 index increased from 1 to 28 after n-3 PUFA supplementation, whereas the number decreased from 2 to 1 after placebo supplementation.

Although fasting glucose and insulin levels did not change during intervention, a slight decrease in plasma GIP was observed within the n-3 PUFA supplemented group. NEFAs, total-cholesterol, LDL-cholesterol and HDL-cholesterol were unaffected by intervention. Fasting TGs significantly decreased after 3-month low-calorie diet (*p* < 0,05) in the whole cohort but the effect was more evident in n-3 PUFA supplemented group. Supplementation with n-3 PUFAs significantly reduced postprandial TG levels, especially in response to high fat meal (HFMTT). This improved lipid tolerance was related to n-3 PUFA supplementation not caloric restriction (Table 2).

**Figure 2:**
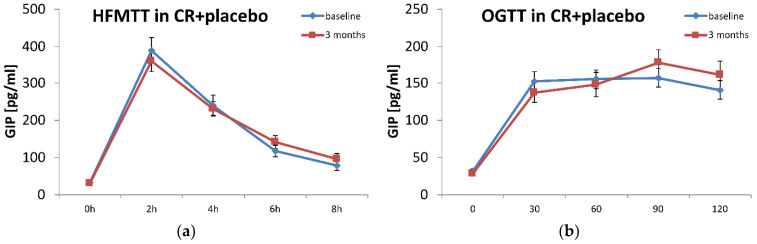

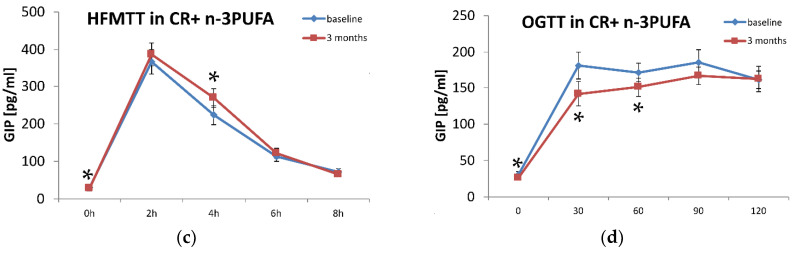
Plasma GIP levels during High Fat mixed Meal Tolerance Test (HFMTT) (**a**,**c**) and Oral Glucose Tolerance Test (OGTT) (**b**,**d**) in plasma of placebo (**a**,**b**) or n-3 PUFA (**c**,**d**) supplemented obese participants. Mean and SEM (bars) presented for baseline (blue line) and after 3-month supplementation (red line). * *p* < 0.05 paired *t*-test. CR—caloric restriction.

Three months of moderate caloric restriction alone did not significantly change plasma GIP concentrations during the 8-h HFMTT (measured in two-hour intervals) or during the OGTT (Figure 2). An addition of 1.8 g/day of n-3 PUFAs for 3 months decreased GIP release in response to glucose, observed 30 min and 60 min after 75 g oral glucose intake (Figure 2).

### 3.3. Effect of n-3 PUFA Supplementation on Carbohydrate and Lipid Metabolism According to GIP Plasma Levels

To find out if there is association of GIP circulating in the blood and the response to n-3 PUFA supplementation, subjects were divided into two subgroups according to fasting GIP plasma level. Patients with fasting GIP level below the median (29 pg/mL) were assigned to the Low GIP group, and those with a fasting GIP level equal to or above the median were assigned to the High GIP group. Anthropometric measures, insulin and blood lipids were not different between these two groups (Table 3). However, fasting glucose was higher in the High GIP group in comparison to Low GIP group (Table 3).

Three months caloric restriction along with supplementation with placebo, besides the reduction in body weight, showed some beneficial metabolic effects such as decreased plasma glucose levels during the OGTT (AUC), and decreased total cholesterol and LDL-cholesterol but only within the Low GIP subgroup (Table 4). Only supplementation of a calorie-restricted diet with n-3 PUFAs improved glucose tolerance (decreased OGTT AUC) in the High GIP subgroup. Although the slight decrease in TGs (fasting or AUC in HFMTT) induced by n-3 PUFA supplementation seemed to be independent of GIP status, the circulating NEFAs significantly decreased in the High GIP group compared to the Low GIP group (Table 4).

### 3.4. Effect of n-3 PUFA supplementation on Plasma Fatty Acid Profile According to Plasma GIP Levels

**Figure 3:**
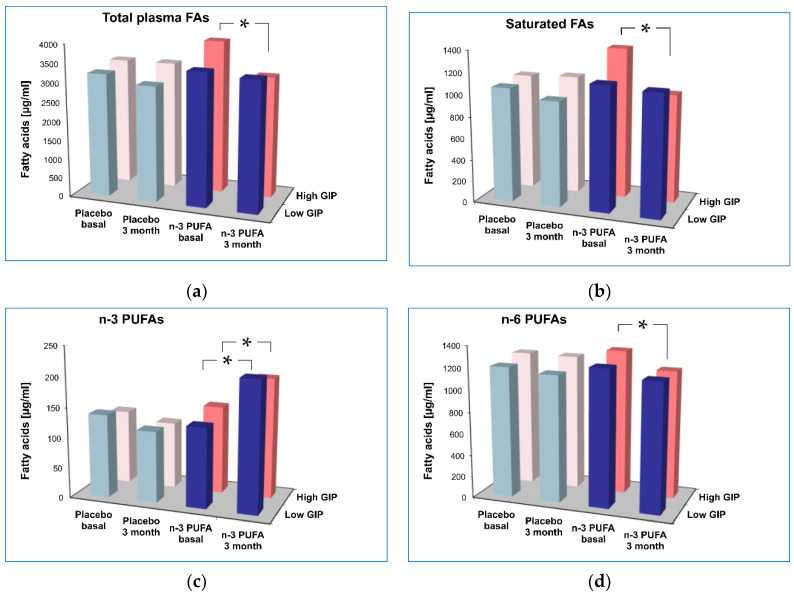
The effect of n-3 PUFA supplementation on total plasma fatty acids (**a**), saturated FAs (**b**), n-3 PUFAs (**c**) and n-6 PUFAs (**d**). * *p* < 0.01 significant change after 3-month intervention vs. baseline.

In the next step, the detailed analysis of different plasma fatty acids was performed in relation to GIP status. We found that moderate caloric restriction alone decreased saturated FA levels in the Low GIP group, in contrast to the High GIP group (Figure 3). The saturated/unsaturated FA ratio also changed differentially depending on GIP status. Supplementation with n-3 PUFAs significantly decreased total plasma fatty acids only in patients with higher plasma GIP levels (Figure 3). Further analysis of the fatty acid profile showed that the changes exclusively in the High GIP n-3 PUFA supplemented group (significant time * GIP group * supplement interactions) concerned saturated FAs (palmitic 16:0, stearic 18:0, myristic (14:0)) as well as unsaturated FAs, but within the last class only monounsaturated FAs and not PUFAs (palmitoleic 16:1n-7, oleic 18:1n-9) were affected (Table 5). In addition, n-3 PUFA supplementation decreased plasma n-6 PUFAs (linoleic 18:2n-6, arachidonic 20:4n-6) within the High GIP group (Table 5).

## 4. Discussion

The omega-3 index is the sum of EPA and DHA in RBC membranes and is expressed as a percentage of total RBC fatty acids. As these long-chain n-3 PUFAs act mainly in the cell membranes and are liberated from cell membranes to interact with signaling pathways and nuclear receptors inside cells, the assessment of omega-3 index has been identified as the most meaningful biomarker of both intake and status of these fatty acids. Omega-3 index is an established risk factor for CHD death, with a cut-off point of 4%, below which there is the greatest risk for CHD death; 4–8% refers to intermediate risk and >8% is regarded as a protective target concentration [20]. N-3 PUFA supplementation (1.8 g/day, DHA:EPA 5:1) resulted in significant increases in the relative contents of DHA and EPA in RBCs, which were closely correlated with changes in plasma concentrations of DHA and EPA. The proportions of individuals whose omega-3 index reached the protective target level after intervention, were 82% after n-3 PUFA supplementation vs. 3% at baseline, compared to 3% after placebo supplementation vs. 7% at baseline.

The low-calorie diet was used to try to decrease body weight of obese participants and indeed it reduced body weight, BMI and body fat, but it did not change any biochemical parameters, except the lowering of plasma TGs. Only the weak effect of CR alone on metabolism observed in our trial may be due to the inclusion and exclusion criteria, as the participants could not have diabetes or hypercholesterolemia or hypertriglyceridemia eligible to pharmacological treatment. The caloric restriction itself was also moderate, and its application time was not long. More evident TG lowering was observed in the group supplemented with n-3 PUFAs, where both fasting and postprandial TGs decreased significantly. As hypertriglyceridemia is a known cardiometabolic risk factor, the effect of n-3 PUFAs is beneficial although the strongest lipid risk factor, LDL-cholesterol, as well as serum total cholesterol, remained unchanged. Other randomized placebo-controlled trials with n-3 PUFAs reported similar results in normolipemic subjects, indicating lowered plasma TG levels and TG-related NMR parameters along with no changes in plasma cholesterol-related parameters, regardless of the proportion of DHA to EPA used [9]. Similarly, after diet supplementation with canned sardine rich in n-3 PUFAs, the serum levels of total cholesterol and HDL-cholesterol did not change, although TG levels showed a trend for a decrease [18]. As reviewed in [21], both EPA and DHA lower TG concentration, with DHA having a greater effect. Simultaneously, total cholesterol levels were unchanged though DHA increased HDL2 subclass and increased LDL-cholesterol concentration and particle size [21]. A meta-analysis of 171 randomized blinded clinical trials with EPA and DHA containing supplements revealed significant mean reductions of TGs of 0.368 mmol/L (CI −0.427 to −0.309) and the effect was dose-dependent [13]. The same analysis indicated a mild increase in both LDL-cholesterol and HDL-cholesterol fractions due to EPA and DHA supplements. This latter effect was not observed in the current study, perhaps due to too small number of participants to detect such a small effect, or due to the relatively low dose of DHA and EPA used (1.8 g/d). The results of the compared study show that the TG response to DHA and EPA supplementation is highly variable among individuals with increased cardiovascular risk and that this response depends on TG and insulin concentrations at baseline [12]. This hypothesis may also apply to our study, as participants were obese, not treated with hypolipemic drugs, and the mean TG concentration in the n-3 PUFA supplemented group was above the target of 1.7 mmol/L.

In our placebo-controlled trial, 3 months of n-3 PUFA supplementation reduced fasting plasma GIP levels as well as postprandial GIP responses to glucose and a high fat meal. To address the question of how n-3 PUFAs can influence circulating GIP levels, it is worth looking at nutrient-induced cellular mechanisms of gut hormone secretion. The gut can assess the nutrient composition of ingested food by nutrient sensing mechanisms in specialized epithelial cells to secrete gut hormones, including GIP, that provide important signals to distinct tissues regulating nutrient metabolism and feeding behavior. The mechanisms include electrogenic transporters, ion channel modulation and nutrient-activated G-protein coupled receptors that converge on the release machinery controlling GIP secretion [22]. Recent studies have shown that n-3 PUFAs have a beneficial effect on the maintenance of proper structure and functioning of membranes of epithelial cells lining the gastrointestinal tract [23]. Considering this fact, the improvement of the gut epithelium by DHA and/or EPA may influence the nutrient-induced cellular mechanism of GIP secretion. Interesting findings regarding a new mechanism of n-3 fatty acid action in the gut have been published recently [24]. Abundant biliary metabolites derived from dietary n-3 PUFAs, N-acyl taurines (NATs), especially DHA-containing NAT, were increased in human and mouse plasma after n-3 PUFA supplementation and potently inhibited intestinal TG hydrolysis and lipid absorption. It could be hypothesized that this n-3 PUFA action leads to weaker stimulation of GIP secretion in intestinal enterocytes, and consequently lower circulating GIP levels, as observed in our placebo-controlled trial.

Fasting as well as postprandial GIP levels are variable among individuals with excess body weight. In some obese subjects, GIP levels are elevated, though its action may be blunted. The reduced action of incretin hormones in type 2 diabetes (T2D) and in obesity is mainly attributed to insensitivity. For example, it was reported that although insulin secretion potentiation with GIP infusion reaches a plateau at ca. 100 pmol/L GIP similarly in glucose tolerant individuals and T2D, GIP stimulated insulin release is reduced by ca. 50% in diabetic patients [25].

As secondary analyses, the changes in plasma fatty acids were assessed in two subgroups using the 29 pg/mL cut-off point for circulating GIP level. Obese patients with relatively high GIP levels benefited from n-3 PUFA supplementation in terms of improved glucose tolerance and decreased circulating NEFA levels. The effects were associated with the normalization of fasting GIP level and with reduced GIP response (at 30 min) to glucose only within the CR + n-3 PUFA HIGH GIP subgroup. The effect was not observed within LOW GIP subgroup supplemented with n-3 PUFA or in HIGH GIP subgroup on CR + placebo. Similarly, some improvements in metabolic risk factors, associated with the reduction in liver enzymes and liver fat content were revealed by meta-analysis of controlled studies on the effectiveness of long-chain n-3 fatty acids in patients with nonalcoholic fatty liver disease (NAFLD) [10]. Our previously published data show an association of high GIP levels in obese subjects with elevated liver enzymes, accompanied by an altered miRNA profile characteristic of hepatic and lipid complications of obesity [16]. Thus, the improvement in insulin sensitivity with a reduction in circulating NEFAs due to n-3 PUFA supplementation observed in our study suggests that there is a reduction in NAFLD risk factors in obese patients with high GIP levels.

Other studies reported either lipolytic or lipogenic action of GIP, depending on the obesity status or glucose tolerance [2,3,4,26,27]. GIP infusion was found to induce lipolysis, shown as increased plasma NEFA and glycerol concentrations, during stable basal insulin substitution and hyperglycemia in men with type 1 diabetes [28]. The results are consistent with the NEFA changes observed in high GIP group after reducing plasma GIP level in our trial. In another study, no acute effects of endogenous GIP released after a standardized fat-rich meal on the postprandial lipoprotein metabolism have been found in overweight/obese men [26]. Especially, production or clearance rate of apoB48 and apoB100 containing TG-rich lipoproteins did not differ between subjects stratified by postprandial GIP levels. In contrast, growing evidence reveals the promotion of fat deposition in tissues by GIP and the blockade of GIP receptor (GIPR) has been proposed to counteract adiposity [2,27]. In type 2 diabetes, a 240-min GIP infusion lowered serum NEFA concentrations, promoting TG accretion in subcutaneous adipose tissue [4]. In an animal model, the administration of peptide based GIPR inhibitors, GIPR antagonists or vaccination against GIP all reduced body weight gain in response to high fat feeding [2]. Moreover, suppression of GIP in an animal model, using innovative K-cell targeted photodynamic therapy, based on the blockade of receptor GPR119 that mediates fatty acid sensing by K cells, led to a decrease in the plasma GIP level and reduction in body weight [29]. Lowering plasma GIP concentration during meal tolerance test in the Mini Egg study was related to improved lipid profile and reduced visceral fat area in overweight /obese men [30]. An interesting explanation of these effects of GIP comes from an experimental study by Killion et al. proving that chronic GIPR agonism desensitizes adipocyte GIPR activity mimicking functional GIPR antagonism [31].

A detailed plasma fatty acids analysis indicated favorable changes in the high GIP group after n-3 PUFA supplementation, in terms of a decreased saturated / unsaturated FA ratio and a reduced amount of particular saturated FAs (myristic, palmitic, stearic) along with an increase in n-3 PUFAs and an n-6 PUFA decrease. Lowering the n-6/ n-3 FA ratio has recently been underlined as an important dietary intervention to prevent LPS-inducible dyslipidemia in an animal model [32]. Thus, the effect observed in obese patients with high GIP levels suggests a beneficial outcome. A significant reduction in the amount of plasma saturated fatty acids may result from reduced absorption due to n-3 PUFA supplementation, which is consistent with lower GIP secretion, because GIP is released in the intestine mainly by saturated FAs [33]. Despite several studies, the role of GIP in lipid metabolism and adipogenesis remain uncertain. Thus, there are still controversies about activation / inhibition of GIPR for the treatment of obesity [3]. GIP is believed to increase TG storage in white adipose tissue not only through stimulating insulin secretion but also by interacting with GIP receptors. In obesity settings, the inhibition of endogenous GIP or its receptor leads to negative energy balance and reduced adiposity, and paradoxically, a similar effect is obtained after administration of exogenous GIP agonists. Proposed mechanisms that explain the anti-obesity effect of supraphysiological dosing of exogenous long-lasting GIP agonists involved direct GIP action in the brain [34]. A study in an animal model revealed that acute administration of acyl-GIP increases cFos neuronal activity in hypothalamic feeding centers, improving glucose handling and decreasing body weight [3]. Recent outcomes from a Phase 2b trial on dual GIP/GLP-1 receptor co-agonist, tirzepatide, indicates its superior efficacy compared to selective GLP-1 agonist or placebo with respect to glycemic control and body weight, associated with lower plasma TGs [35]. Post hoc explanatory lipidomic analyses revealed a change in TG and diglyceride FA content with the bias toward shorter and saturated FAs [36]. This finding may explain the relationship of plasma GIP and saturated /unsaturated FA ratio observed in our study, as well as more potent decrease in particular saturated FAs with simultaneous decrease in plasma GIP levels during intervention.

Other gut hormones, which activity may be disturbed in obesity, also play a role in regulating lipids profile. Glucagon-like peptide-1 (GLP-1), the incretin which has received the most attention in decades, mainly due to critical role in glycemic control, in its multidirectional action also influences lipid metabolism. Nevertheless, most of the reports on the improvement of the lipid profile, including modest reductions in LDL-C, total cholesterol, and triglycerides, come from clinical trials of GLP-1R agonists in patients with type 2 diabetes and the observed effects were exerted by exogenous, long-acting GLP-1 [37] (Sun 2015). Long-chain fatty acids stimulate secretion of glucagon-like peptide-1 (GLP-1) through free fatty acids receptor 1 (FFA1), though the activation is probably not sufficient to induce a pronounced incretin response. Moreover, in humans, the ingestion of polyunsaturated fat induces a weaker GLP-1 response than ingestion of monounsaturated or saturated fat [38] (Hjørne 2022).

## 5. Conclusions

In conclusion, three months caloric restriction improved some metabolic parameters in obese patients with lower (<29 pg/mL) plasma GIP level but did not influence these same metabolic parameters in obese patients with higher GIP level, which may reflect early metabolic complications in obesity. However, the addition of n-3 PUFA supplementation significantly improved the plasma lipid profile in obese patients with high plasma GIP level. Plasma GIP level may indicate subjects who would potentially benefit from n-3 PUFA supplementation.

## Figures and Tables

**Table 1:** Characteristics of participants at baseline.

	CR + PLACEBO n = 28	CR + n-3 PUFAs n = 34	*p*
	Mean (SD)	Mean (SD)	
Age [years]	47.7 (12.9)	47.4 (10.7)	ns
Sex [women]	24 (86%)	22 (65%)	ns
Weight [kg]	93.47 (13.23)	94.19 (16.83)	ns
BMI [kg/m^2^]	34.35 (4.18)	32.98 (4.35)	ns
Body fat [%]	40.61 (5.14)	37.29 (6.04)	ns
Glucose [mmol/L]	5.18 (0.72)	5.32 (0.61)	ns
Insulin [µIU/mL]	16.19 (9.27)	14.89 (9.26)	ns
GIP [pg/mL]	32.21 (20.57)	30.47 (15.15)	ns
NEFAs [mmol/L]	0.78 (0.27)	0.78 (0.32)	ns
TGs [mmol/L]	1.41 (0.66)	1.89 (1.12)	ns
Total-cholesterol [mmol/L]	5.37 (1.08)	5.67 (0.92)	ns
HDL-cholesterol [mmol/L]	1.34 (0.25)	1.25 (0.23)	ns
LDL-cholesterol [mmol/L]	3.39 (0.90)	3.65 (0.92)	ns

Data presented as mean (SD), except sex presented as number (percentage); ns—*p* > 0.05 *t*-test; BMI—body mass index, GIP—glucose-dependent insulinotropic polypeptide, NEFAs—non-esterified fatty acids, TGs—triglycerides.

**Table 2:** Effect of placebo and n-3 PUFA supplementation on anthropometrics, EPA and DHA status and biochemical parameters.

	CR + PLACEBO (n = 28)		CR + n-3 PUFAs (n = 34)		
	Baseline	3-Months	Baseline	3-Months	Time * Suppl. Interaction
	Mean (SD)	Mean (SD)	Mean (SD)	Mean (SD)	*p*
Weight [kg]	93.47	86.30 **	94.19	88.24 **	0.244
	(13.23)	(13.77)	(16.83)	(15.69)	
BMI [kg/m^2^]	34.35	31.73 *	32.98	30.81 **	0.222
	(4.18)	(4.66)	(4.35)	(4.07)	
Body fat [%]	40.61	37.60 *	37.29	34.05 **	0.806
	(5.14)	(6.14)	(6.04)	(7.62)	
EPA in plasma	42.67	32.16 *	41.63	67.36 **	<0.001
[µg/mL]	(24.97)	(12.80)	(21.83)	(32.18)	
DHA in plasma	68.09	62.24	67.06	119.33 **	<0.001
[µg/mL]	(27.99)	(19.14)	(23.58)	(30.75)	
EPA in RBCs	1.16	0.98 *	1.06	1.97 **	<0.001
[%]	(0.46)	(0.36)	(0.37)	(0.84)	
DHA in RBC m.	4.45	4.44	4.31	7.53 **	<0.001
[%]	(1.17)	(1.18)	(0.95)	(1.10)	
Omega-3 index	5.61	5.42	5.37	9.49 **	<0.001
[%]	(1.51)	(1.47)	(1.22)	(1.71)	
Glucose fasting	5.18	5.15	5.32	5.24	0.348
[mmol/L]	(0.72)	(0.51)	(0.61)	(0.55)	
Insulin fasting	16.19	14.62	14.89	13.60	0.584
[µIU/mL]	(9.27)	(8.76)	(9.26)	(5.33)	
GIP fasting	32.21	30.65	30.47	26.61 ^#^	0.226
[pg/mL]	(20.57)	(18.05)	(15.15)	(26.05)	
GIP AUC-OGTT [pg/mL*min]	76,586 (34,902)	70,425 (33,541)	42,152 (38,120)	68,783 (36,920)	0.702
GIP AUC-HFMTT [pg/mL*min]	399,913 (145,822)	429,312 (182,117)	420,605 (202,863)	427,395 (185,684)	0.676
NEFA fasting	0.78	0.83	0.78	0.74	0.384
[mmol/L]	(0.27)	(0.37)	(0.32)	(0.27)	
TG fasting	1.41	1.26	1.89	1.44 *	0.134
[mmol/L]	(0.66)	(0.48)	(1.12)	(0.66)	
TG AUC-OGTT [mmol/L*min]	612.97 (350.62)	606.06 (353.74)	674.44 (394.90)	596.40 * (312.69)	0.393
TG AUC-HFMTT [mmol/L*min]	3546.62 (1843.27)	3214.71 (1448.03)	4823.93 (2460.67)	3520.14 * (1428.75)	0.023
Total-cholesterol	5.37	5.15	5.67	5.37	0.779
[mmol/L]	(1.08)	(1.24)	(0.92)	(1.03)	
HDL-cholesterol	1.34	1.35	1.25	1.25	0.739
[mmol/L]	(0.25)	(0.27)	(0.23)	(0.18)	
LDL-cholesterol	3.39	3.23	3.65	3.47	0.619
[mmol/L]	(0.90)	(1.05)	(0.92)	(1.01)	

Data are presented as mean (SD); ANOVA for repeated measurements with Bonferroni post-hoc test were used for analysis of supplementation effects by time and group (suppl. placebo vs. n-3PUFA) interaction; * for *p* < 0.05, ** for *p* < 0.001 significant change after 3 month intervention vs baseline; ^#^ for *p* < 0.05 *t*-test for repeated measurements in analysis of time effect within group. Abbreviations: AUC—area under curve, OGTT—oral glucose tolerance test, HFMTT—high fat mixed meal tolerance test; NEFAs—non-esterified fatty acids; RBC—red blood cell membranes.

**Table 3:** Characteristics of participants according plasma GIP level at baseline.

	LOW GIP n = 32	HIGH GIP n = 30	
	Mean (SD)	Mean (SD)	*p*
Weight [kg]	94.28 (14.18)	93.75 (16.71)	n.s.
BMI [kg/m^2^]	33.91 (4.08)	33.42 (4.64)	n.s.
Body fat [%]	39.21 (6.06)	38.30 (5.77)	n.s.
Glucose [mmol/L]	5.07 (0.49)	5.8 (0.67)	0.039
Insulin [µIU/mL]	13.80 (7.84)	17.31 (10.30)	n.s.
GIP [pg/mL]	17.97 (7.23)	44.75 (19.06)	<0.001
NEFAs [mmol/L]	0.83 (0.33)	0.72 (0.26)	n.s.
TGs [mmol/L]	1.67 (1.05)	1.64 (0.89)	n.s.
Total-cholesterol [mmol/L]	5.51 (0.96)	5.61 (1.05)	n.s.
HDL-cholesterol [mmol/L]	1.34 (0.24)	1.25 (0.23)	n.s.
LDL-cholesterol [mmol/L]	3.48 (0.93)	3.64 (0.89)	n.s.

Data presented as mean (SD). LOW GIP—group presenting plasma GIP level < 29 pg/mL, HIGH GIP—group presenting plasma GIP level > 29 pg/mL; BMI—body mass index, GIP—glucose-dependent insulinotropic polypeptide, NEFAs—non-esterified fatty acids, TGs—triglycerides.

**Table 4:** Effect of placebo and n-3 PUFA supplementation on anthropometrics, EPA and DHA status and biochemical parameters according to GIP plasma levels.

	CR + PLACEBO (n = 28)					CR + n-3 PUFA (n = 34)						
	LOW GIP (n = 13)		HIGH GIP (n = 15)		Time * GIP Group Interaction	LOW GIP (19)		HIGH GIP (n = 15)		Time * GIP Group Interaction	Time * Supplementation Interaction	Time * GIP Group * Supplementation Interaction
	Baseline	3-Months	Baseline	3-Months		Baseline	3-Months	Baseline	3-Months			
	Mean (SD)	Mean (SD)	Mean (SD)	Mean (SD)	*p*	Mean (SD)	Mean (SD)	Mean (SD)	Mean (SD)	*p*	*p*	*p*
Weight [kg]	93.28	86.10 **	94.29	86.59 **	0.771	94.96	89.25 **	93.21	86.96 **	0.691	0.180	0.994
	(11.61)	(12.25)	(15.49)	(16.14)		(15.97)	(14.84)	(18.39)	(17.14)			
BMI [kg/m^2^]	34.34	31.69 **	34.67	31.89 **	0.819	33.62	31.61 **	32.17	29.79 **	0.426	0.165	0.757
	(3.47)	(3.89)	(4.92)	(5.57)		(4.51)	(4.30)	(4.14)	(3.66)			
EPA in RBC membrane	1.26	1.06	1.09	0.93	0.804	1.03	1.94 **	1.10	2.01 **	0.982	<0.001	0.913
[%]	(0.52)	(0.46)	(0.41)	(0.29)		(0.37)	(0.86)	(0.39)	(0.83)			
DHA in RBC membrane	4.74	4.69	4.43	4.34	0.919	4.47	7.56 **	4.12	7.48 **	0.531	<0.001	0.598
[%]	(1.20)	(1.39)	(1.06)	(1.03)		(0.97)	(1.08)	(0.90)	(1.17)			
Omega-3 index	5.99	5.75	5.52	5.27	0.979	5.50	9.50 **	5.22	9.48 **	0.662	<0.001	0.717
[%]	(1.61)	(1.73)	(1.34)	(1.27)		(1.29)	(1.76)	(1.14)	(1.70)			
Glucose fasting	4.93	4.95	5.23	5.32	0.739	5.16	5.18	5.53	5.33	0.294	0.315	0.322
[mmol/L]	(0.48)	(0.45)	(0.65)	(0.52)		(0.50)	(0.57)	(0.68)	(0.53)			
Glucose OGTT AUC	3767	3273 *	3388	3636	0.005	3288	3250	3821	3491 *	0.207	0.716	0.003
[mmol/L * min]	(716)	(647)	(503)	(951)		(800)	(828)	(916)	(869)			
Insulin fasting	14.34	12.49	17.79	16.47	0.811	13.42	13.30	16.79	13.96	0.848	0.597	0.760
[µIU/mL]	(9.93)	(8.51)	(8.68)	(8.83)		(6.22)	(4.58)	(12.12)	(6.29)			
Insulin OGTT AUC	45,312	32,581	48,570	50,271	0.119	42,748	36,063	45,070	31,547	0.942	0.579	0.246
[µIU/mL * min]	(31,114)	(21,955)	(25,845)	(34,038)		(22,599)	(21,120)	(41,210)	(16,544)			
GIP fasting	18.25	18.72	42.59	35.69	0.364	17.77	18.73	46.90	35.82 ^#^	0.094	0.728	0.659
[pg/mL]	(7.76)	(10.89)	(13.40)	(21.40)		(7.05)	(11.07)	(23.72)	(22.42)			
GIP 30′ OGTT	150.1	134.2	154.9	141.0	0.942	153.2	122.5	215.7	166.7 ^#^	0.432	0.148	0.558
[pg/mL]	(62.2)	(68.3)	(77.1)	(73.7)		(58.9)	(58.8)	(145.4)	(131.2)			
GIP OGTT AUC	59,660	60,106	71,837	72,937	0.947	65,736	58,639	89,320	76,606	0.514	0.123	0.453
[pg/mL * min]	(19,250)	(29,132)	26,553)	(39,108)		(22,474)	(27,020)	(43,708)	(34,523)			
TG fasting	1.26	1.04	1.48	1.48	0.171	1.96	1.42	1.80	1.46	0.412	0.096	0.735
[mmol/L]	(0.63)	(0.29)	(0.71)	(0.56)		(1.20)	(0.60)	(1.04)	(0.75)			
TG OGTT AUC	3353	2796	3842	3544	0.451	4919	3448	4703	3608	0.381	0.069	0.924
[mmol/L *min]	(1552)	(1081)	(2246)	(1738)		(2673)	(1434)	(22,490)	(1476)			
NEFAs fasting	0.670	0.663	0.767	0.744	0.959	0.732	0.733	0.750	0.615 *	0.028	0.681	0.179
[mmol/L]	(0.253)	(0.266)	(0.424)	(0.399)		(0.251)	(0.280)	(0.352)	(0.229)			
NEFAs OGTT AUC	1411	1386	1618	1492	0.519	1665	1514	1565	1286 *	0.111	0.409	0.670
[mmol/L * min]	(445)	(389)	(735)	(504)		(317)	(372)	(646)	(510)			
Total-cholesterol	5.45	5.00 *	5.40	5.35	0.091	5.55	5.32	5.82	5.43	0.938	0.906	0.211
[mmol/L]	(1.06)	(1.19)	(1.14)	(1.35)		(0.92)	(1.14)	(0.93)	(0.92)			
HDL-cholesterol	1.44	1.39	1.26	1.35	0.087	1.27	1.26	1.23	1.23	0.999	0.646	0.194
[mmol/L]	(0.26)	(0.22)	(0.23)	(0.31)		(0.22)	(0.21)	(0.24)	(0.15)			
LDL-cholesterol	3.44	3.14 *	3.46	3.32	0.297	3.50	3.42	3.84	3.53	0.942	0.4312	0.437
[mmol/L]	(0.90)	(1.02)	(0.93)	(1.14)		(0.98)	(1.13)	(0.83)	(0.89)			

Data presented as mean (SD); ANOVA for repeated measurements with post-hoc test Tukey HSD were used for analyses of GIP group effect within intervention groups and for analysis of supplementation effect within the whole cohort. * for *p* < 0.05, ** *p* < 0.01 significant change after 3 month intervention vs. baseline. ^#^ *p* < 0.05 within group *t*-Test with repeated measurements.

**Table 5:** Effect of placebo and n-3 PUFA supplementation on plasma fatty acids profile according to GIP plasma levels.

	CR + PLACEBO (n = 28)					CR + n-3 PUFA (n = 34)						
	LOW GIP (n = 13)		HIGH GIP (n = 15)		Time * GIP Group Interaction	LOW GIP (19)		HIGH GIP (n = 15)		Time * GIP Group Interaction	Time * Supplementation Interaction	Time * GIP Group * Supplementation Interaction
	Baseline	3-Months	Baseline	3-Months		Baseline	3-Months	Baseline	3-Months			
	Mean (SD)	Mean (SD)	Mean (SD)	Mean (SD)	*p*	Mean (SD)	Mean (SD)	Mean (SD)	Mean (SD)	*p*	*p*	*p*
Total FAs	3223	3007	3342	3344	0.191	3453	3364	3996	3162 **	0.006	0.027	0.003
[µg/mL]	(507)	(490)	(817)	(828)		(519)	(579)	(1194)	(637)			
Saturated FAs	1057	972 *	1083	1102	0.079	1150	1119	1390	1001 **	0.004	0.011	0.001
[µg/mL]	(176)	(159)	(279)	(287)		(225)	(239)	(520)	(217)			
Myristic acid (14:0)	36.50	29.37	35.21	40.66	0.005	44.91	38.95	65.46	29.32 **	0.018	0.004	0.003
[µg/mL]	(13.78)	(12.03)	(16.08)	(18.52)		(17.22)	(18.68)	(49.78)	(9.77)			
Palmitic acid (16:0)	729.3	678.5	763.6	775.1	0.177	805.8	791.8	985.2	706.8 **	0.003	0.013	0.002
[µg/mL]	(118.0)	(107.2)	(205.3)	(199.0)		(164.1)	(171.7)	(384.6)	(163.1)			
Stearic acid (18:0)	239.5	219.1	235.8	238.2	0.055	249.96	240.46	286.92	217.00 **	0.008	0.021	0.002
[µg/mL]	(42.7)	(39.3)	(57.0)	(65.4)		(46.45)	(51.01)	(91.26)	(42.86)			
Unsaturated FAs	2167	2035	2259	2243	0.298	2304	2245	2606	2161 **	0.012	0.061	0.009
[µg/mL]	(345)	(338)	(547)	(547)		(319)	(361)	(684)	(423)			
Saturated /unsaturated	0.489	0.479	0.479	0.491	0.016	0.498	0.497	0.521	0.462 **	0.011	0.015	0.002
FAs [µg/mL]	(0.037)	(0.029)	(0.034)	(0.028)		(0.051)	(0.056)	(0.068)	(0.021)			
MUFAs	825.2	754.2	882.6	885.5	0.257	918.9	857.1	1135.0	789.8 **	0.009	0.009	0.006
[µg/mL]	(144)	(128)	(294)	(292)		(220.6)	(222.6)	(466.1)	(209.0)			
Palmitoleic acid (16:1n-7)	108.2	91.1	103.3	102.9	0.136	108.1	98.9	126.0	78.4 **	0.024	0.057	0.009
[µg/mL]	(31.8)	(25.6)	(41.4)	(39.1)		(33.5)	(47.0)	(59.0)	(36.1)			
Oleic acid (18:1n-9)	671.8	616.8	734.8	740.1	0.265	770.1	713.4	966.7	664.9 **	0.012	0.008	0.008
[µg/mL]	(117.7)	(108.7)	(250.6)	(256.3)		(196.1)	(184.5)	(428.1)	(181.3)			
Nervonic acid (24:1)	45.20	46.40	44.60	42.5	0.286	40.66	44.73	42.32	46.42	0.993	0.021	0.391
[µg/mL]	(7.3)	(7.2)	(16.3)	(9.5)		(6.04)	(7.97)	(8.85)	(9.19)			
PUFAs	1341	1280	1376	1357	0.491	1385	1388	1471	1371	0.082	0.834	0.086
[µg/mL]	(227)	(228)	(277)	(291)		(180)	(211)	(240)	(239)			
n-3 PUFAs	137.2	117.3	123.3	110.4	0.633	131.7	213.2 **	144.8	197.6 *	0.208	<0.001	0.202
[µg/mL]	(50.4)	(46.2)	(45.2)	(21.4)		(45.8)	(73.9)	(54.6)	(33.2)			
n-6 PUFAs	1204	1163	1253	1247	0.509	1253	1175	1326	1173 **	0.162	0.015	0.145
[µg/mL]	(220)	(200)	(246)	(277)		(174)	(194)	(200)	(222)			
n-3/n-6 ratio	0.117	0.100	0.097	0.090	0.404	0.107	0.186 **	0.107	0.172 **	0.495	<0.001	0.329
[µg/mL]	(0.046)	(0.033)	(0.027)	(0.015)		(0.042)	(0.069)	(0.033)	(0.033)			
Essential FAs	946	902	993	1003	0.245	999	932	1061	930 *	0.204	0.018	0.087
[µg/mL]	(199)	(194)	(190)	(226)		(144)	(179)	(173)	(201)			
Linolenic acid (18:3n-3)	22.02	17.70	22.46	22.08	0.263	25.21	20.01	33.34	19.30 *	0.104	0.030	0.056
[µg/mL]	(11.43)	(7.65)	(10.00)	(9.18)		(11.01)	(7.27)	(23.87)	(7.68)			
Eicosapentaenoic acid	43.54	33.66	37.56	29.81	0.774	38.56	70.05 **	45.58	63.90 *	0.263	<0.001	0.287
(20:5n-3) [µg/mL]	(23.08)	(17.92)	(18.14)	(6.43)		(18.86)	(41.43)	(25.33)	(14.21)			
Docosahexaenoic acid	71.62	65.92	63.27	58.46	0.899	67.98	123.17 **	65.89	114.41 **	0.562	<0.001	0.589
(22:6n-6) [µg/mL]	(25.22)	(24.04)	(26.44)	(12.37)		(22.61)	(34.91)	(25.59)	(24.79)			
Linoleic acid (18:2n-6)	924.4	884.6	970.3	981.4	0.262	973.8	911.5	1027.4	910.8 **	0.246	0.024	0.108
[µg/mL]	(193.6)	(188.5)	(183.6)	(220.5)		(143.8)	(176.1)	(156.1)	(197.6)			
Arachidonic acid	279.7	278.8	282.7	265.4	0.378	279.4	263.2	299.1	262.5 *	0.186	0.075	0.365
(20:4n-6) [µg/mL]	(41.2)	(33.5)	(102.4)	(69.0)		(48.3)	(49.5)	(81.9)	(56.7)			

Data presented as mean (SD); ANOVA for repeated measurements with Bonferroni post-hoc test were used for analyses of GIP group effect within intervention groups and for analysis of supplementation effect within the whole cohort. * *p* < 0.05, ** *p* < 0.01 significant change after 3-month intervention vs. baseline.

## Data Availability

The data presented in this study can be made available by contacting the corresponding author.

## References

[1] Samms R.J., Coghlan M.P., Sloop K.W. (2020). How May GIP Enhance the Therapeutic Efficacy of GLP-1?. Trends Endocrinol. Metab..

[2] Irwin N., Gault V.A., O’Harte F.P.M., Flatt P.R. (2020). Blockade of Gastric Inhibitory Polypeptide (GIP) Action as a Novel Means of Countering Insulin Resistance in the Treatment of Obesity-Diabetes. Peptides.

[3] Zhang Q., Delessa C.T., Augustin R., Bakhti M., Colldén G., Drucker D.J., Feuchtinger A., Caceres C.G., Grandl G., Harger A. (2021). The Glucose-Dependent Insulinotropic Polypeptide (GIP) Regulates Body Weight and Food Intake via CNS-GIPR Signaling. Cell Metab..

[4] Thondam S.K., Daousi C., Wilding J.P.H., Holst J.J., Ameen G.I., Yang C., Whitmore C., Mora S., Cuthbertson D.J. (2017). Glucose-Dependent Insulinotropic Polypeptide Promotes Lipid Deposition in Subcutaneous Adipocytes in Obese Type 2 Diabetes Patients: A Maladaptive Response. Am. J. Physiol.-Endocrinol. Metab..

[5] Fritsche L., Heni M., Eckstein S.S., Hummel J., Schürmann A., Häring H.-U., Preißl H., Birkenfeld A.L., Peter A., Fritsche A. (2022). Incretin Hypersecretion in Gestational Diabetes Mellitus. J. Clin. Endocrinol. Metab..

[6] Polus A., Zapala B., Razny U., Gielicz A., Kiec-Wilk B., Malczewska-Malec M., Sanak M., Childs C.E., Calder P.C., Dembinska-Kiec A. (2016). Omega-3 Fatty Acid Supplementation Influences the Whole Blood Transcriptome in Women with Obesity, Associated with pro-Resolving Lipid Mediator Production. Biochim. Biophys. Acta-Mol. Cell Biol. Lipids.

[7] Razny U., Goralska J., Calder P.C., Gruca A., Childs C.E., Kapusta M., Slowinska-Solnica K., Dembinska-Kiec A., Solnica B., Malczewska-Malec M. (2021). The Effect of Caloric Restriction with and without N-3 Pufa Supplementation on Bone Turnover Markers in Blood of Subjects with Abdominal Obesity: A Randomized Placebo-Controlled Trial. Nutrients.

[8] del Gobbo L.C., Imamura F., Aslibekyan S., Marklund M., Virtanen J.K., Wennberg M., Yakoob M.Y., Chiuve S.E., dela Cruz L., Frazier-Wood A.C. (2016). ω-3 Polyunsaturated Fatty Acid Biomarkers and Coronary Heart Disease: Pooling Project of 19 Cohort Studies. JAMA Intern. Med..

[9] Yang Z.H., Amar M., Sampson M., Courville A.B., Sorokin A.V., Gordon S.M., Aponte A.M., Stagliano M., Playford M.P., Fu Y.P. (2020). Comparison of Omega-3 Eicosapentaenoic Acid versus Docosahexaenoic Acid-Rich Fish Oil Supplementation on Plasma Lipids and Lipoproteins in Normolipidemic Adults. Nutrients.

[10] Musa-Veloso K., Venditti C., Lee H.Y., Darch M., Floyd S., West S., Simon R. (2018). Systematic Review and Meta-Analysis of Controlled Intervention Studies on the Effectiveness of Long-Chain Omega-3 Fatty Acids in Patients with Nonalcoholic Fatty Liver Disease. Nutr. Rev..

[11] Hodson L., Gunn P.J. (2019). The Regulation of Hepatic Fatty Acid Synthesis and Partitioning: The Effect of Nutritional State. Nat. Rev. Endocrinol..

[12] Allaire J., Vors C., Harris W.S., Jackson K.H., Tchernof A., Couture P., Lamarche B. (2019). Comparing the Serum TAG Response to High-Dose Supplementation of Either DHA or EPA among Individuals with Increased Cardiovascular Risk: The ComparED Study. Br. J. Nutr..

[13] AbuMweis S., Jew S., Tayyem R., Agraib L. (2018). Eicosapentaenoic Acid and Docosahexaenoic Acid Containing Supplements Modulate Risk Factors for Cardiovascular Disease: A Meta-Analysis of Randomised Placebo-Control Human Clinical Trials. J. Hum. Nutr. Diet..

[14] Calder P.C. (2022). Omega-3 Fatty Acids and Metabolic Partitioning of Fatty Acids within the Liver in the Context of Nonalcoholic Fatty Liver Disease. Curr. Opin. Clin. Nutr. Metab. Care.

[15] Razny U., Kiec-Wilk B., Polus A., Goralska J., Malczewska-Malec M., Wnek D., Zdzienicka A., Gruca A., Childs C.E., Kapusta M. (2015). Effect of Caloric Restriction with or without N-3 Polyunsaturated Fatty Acids on Insulin Sensitivity in Obese Subjects: A Randomized Placebo Controlled Trial. BBA Clin..

[16] Góralska J., Raźny U., Polus A., Dziewońska A., Gruca A., Zdzienicka A., Dembińska-Kieć A., Solnica B., Micek A., Kapusta M. (2020). Enhanced GIP Secretion in Obesity Is Associated with Biochemical Alteration and MiRNA Contribution to the Development of Liver Steatosis. Nutrients.

[17] Razny U., Goralska J., Zdzienicka A., Gruca A., Zapala B., Micek A., Dembinska-Kiec A., Solnica B., Malczewska-Malec M. (2018). High Fat Mixed Meal Tolerance Test Leads to Suppression of Osteocalcin Decrease in Obese Insulin Resistant Subjects Compared to Healthy Adults. Nutrients.

[18] Bandarra N.M., Palma P., Batista I., Nunes M.L., Morais G., Bruges M., Dickson J., Barata J.D., Silva-Lima B. (2002). Effect of a Supplemented Diet with Canned Sardine on the Lipid Fraction of Human Plasma and Erythrocytes. J. Aquat. Food Prod. Technol..

[19] Glaser C., Demmelmair H., Koletzko B. (2010). High-Throughput Analysis of Total Plasma Fatty Acid Composition with Direct in Situ Transesterification. PLoS ONE.

[20] Harris W.S. (2008). The Omega-3 Index as a Risk Factor for Coronary Heart Disease. Am. J. Clin. Nutr..

[21] Innes J.K., Calder P.C. (2018). The Differential Effects of Eicosapentaenoic Acid and Docosahexaenoic Acid on Cardiometabolic Risk Factors: A Systematic Review. Int. J. Mol. Sci..

[22] Lu V.B., Gribble F.M., Reimann F. (2021). Nutrient-Induced Cellular Mechanisms of Gut Hormone Secretion. Nutrients.

[23] Durkin L.A., Childs C.E., Calder P.C. (2021). Omega-3 Polyunsaturated Fatty Acids and the Intestinal Epithelium—A Review. Foods.

[24] Grevengoed T.J., Trammell S.A.J., Svenningsen J.S., Makarov M.V., Nielsen T.S., Jacobsen J.C.B., Treebak J.T., Calder P.C., Migaud M.E., Cravatt B.F. (2021). An Abundant Biliary Metabolite Derived from Dietary Omega-3 Polyunsaturated Fatty Acids Regulates Triglycerides. J. Clin. Investig..

[25] Grespan E., Giorgino T., Natali A., Ferrannini E., Mari A. (2021). Different Mechanisms of GIP and GLP-1 Action Explain Their Different Therapeutic Efficacy in Type 2 Diabetes. Metab. Clin. Exp..

[26] Taskinen M.-R., Matikainen N., Björnson E., Söderlund S., Ainola M., Hakkarainen A., Lundbom N., Sihlbom C., Thorsell A., Andersson L. (2022). Role of Endogenous Incretins in the Regulation of Postprandial Lipoprotein Metabolism. Eur. J. Endocrinol..

[27] Heimbürger S.M., Bergmann N.C., Augustin R., Gasbjerg L.S., Christensen M.B., Knop F.K. (2020). Glucose-Dependent Insulinotropic Polypeptide (GIP) and Cardiovascular Disease. Peptides.

[28] Heimburger S.M.N., Nielsen C.N., Calanna S., Holst J.J., Vilsbøll T., Knop F.K., Christensen M.B. (2022). Glucose-Dependent Insulinotropic Polypeptide Induces Lipolysis during Stable Basal Insulin Substitution and Hyperglycaemia in Men with Type 1 Diabetes: A Randomized, Double-Blind, Placebo-Controlled, Crossover Clinical Trial. Diabetes Obes. Metab..

[29] Lee S., Na K. (2020). Lipid Photosensitizers for Suppression of Gastric Inhibitory Polypeptide in Obese with Type 2 Diabetes. Biomaterials.

[30] Sakane N., Osaki N., Takase H., Suzuki J., Suzukamo C., Nirengi S., Suganuma A., Shimotoyodome A. (2019). The Study of Metabolic Improvement by Nutritional Intervention Controlling Endogenous GIP (Mini Egg Study): A Randomized, Cross-over Study. Nutr. J..

[31] Killion E.A., Chen M., Falsey J.R., Sivits G., Hager T., Atangan L., Helmering J., Lee J., Li H., Wu B. (2020). Chronic Glucose-Dependent Insulinotropic Polypeptide Receptor (GIPR) Agonism Desensitizes Adipocyte GIPR Activity Mimicking Functional GIPR Antagonism. Nat. Commun..

[32] Park S., Lee J.-J., Lee J., Lee J.K., Byun J., Kim I., Ha J.-H. (2022). Lowering N-6/n-3 Ratio as an Important Dietary Intervention to Prevent LPS-Inducible Dyslipidemia and Hepatic Abnormalities in Ob/Ob Mice. Int. J. Mol. Sci..

[33] Itoh K., Moriguchi R., Yamada Y., Fujita M., Yamato T., Oumi M., Holst J.J., Seino Y. (2014). High Saturated Fatty Acid Intake Induces Insulin Secretion by Elevating Gastric Inhibitory Polypeptide Levels in Healthy Individuals. Nutr. Res..

[34] Fukuda M. (2021). The Role of GIP Receptor in the CNS for the Pathogenesis of Obesity. Diabetes.

[35] Nauck M.A., Quast D.R., Wefers J., Pfeiffer A.F.H. (2021). The Evolving Story of Incretins (GIP and GLP-1) in Metabolic and Cardiovascular Disease: A Pathophysiological Update. Diabetes Obes. Metab..

[36] Pirro V., Roth K.D., Lin Y., Willency J.A., Milligan P.L., Wilson J.M., Ruotolo G., Haupt A., Newgard C.B., Duffin K.L. (2022). Effects of Tirzepatide, a Dual GIP and GLP-1 RA, on Lipid and Metabolite Profiles in Subjects with Type 2 Diabetes. J. Clin. Endocrinol. Metab..

[37] Sun F., Wu S., Wang J., Guo S., Chai S., Yang Z., Li S., Zhang Y., Ji L., Zhan S. (2015). Effect of Glucagon-like Peptide-1 Receptor Agonists on Lipid Profiles among Type 2 Diabetes: A Systematic Review and Network Meta-Analysis. Clin. Ther..

[38] Hjørne A.P., Modvig I.M., Holst J.J. (2022). The Sensory Mechanisms of Nutrient-Induced GLP-1 Secretion. Metabolites.

